# Regulation of cerebral cortex development by Rho GTPases: insights from *in vivo* studies

**DOI:** 10.3389/fncel.2014.00445

**Published:** 2015-01-07

**Authors:** Roberta Azzarelli, Thomas Kerloch, Emilie Pacary

**Affiliations:** ^1^Department of Oncology, Hutchison/MRC Research Centre, Cambridge Biomedical Campus, University of CambridgeCambridge, UK; ^2^Institut National de la Santé et de la Recherche Médicale U862, Neurocentre MagendieBordeaux, France; ^3^Institut National de la Santé et de la Recherche Médicale, Physiopathologie de la Plasticité Neuronale, Université de BordeauxBordeaux, France

**Keywords:** Rho GTPases, cerebral cortex, neuronal development, cortical malformations, GAP, GEF

## Abstract

The cerebral cortex is the site of higher human cognitive and motor functions. Histologically, it is organized into six horizontal layers, each containing unique populations of molecularly and functionally distinct excitatory projection neurons and inhibitory interneurons. The stereotyped cellular distribution of cortical neurons is crucial for the formation of functional neural circuits and it is predominantly established during embryonic development. Cortical neuron development is a multiphasic process characterized by sequential steps of neural progenitor proliferation, cell cycle exit, neuroblast migration and neuronal differentiation. This series of events requires an extensive and dynamic remodeling of the cell cytoskeleton at each step of the process. As major regulators of the cytoskeleton, the family of small Rho GTPases has been shown to play essential functions in cerebral cortex development. Here we review *in vivo* findings that support the contribution of Rho GTPases to cortical projection neuron development and we address their involvement in the etiology of cerebral cortex malformations.

## Introduction

In humans, the cerebral cortex is responsible for the processes of thought, perception and memory and serves as the seat of advanced motor functions, social abilities and language. These functions relies on the proper development of two main populations of neurons, projection or pyramidal neurons, which are glutamatergic and excitatory, and interneurons, which are GABAergic and inhibitory. Neurons in the cerebral cortex are arranged into six distinct layers different in terms of connectivity, gene expression profile and birthdate (Molyneaux et al., 2007). Projection neurons originate from progenitors located in the cortex whereas interneurons are born in the ventral domains of the telencephalon and then migrate tangentially to reach the cortex (Kriegstein and Noctor, 2004).

After closure of the neural tube, the epithelium lining the ventricles becomes a specialized neuroepithelium. It consists of a single sheet of progenitor cells, called neuroepithelial cells (NEs). At early developmental stages, around E10 in mouse, NEs along the dorsal surface of lateral ventricles self-renew to expand the progenitor pool and then convert into cells expressing glial markers such as the astrocyte-specific glutamate transporter (GLAST) and brain lipid-binding protein (BLBP), the radial glial cells (RGs). The asymmetric divisions of these RGs are responsible for producing cortical projection neurons either directly or indirectly through intermediate progenitor cells (IPs) or outer radial glial cells (oRGs). Newborn projections neurons then migrate radially in a step-wise fashion to their final destination using RG fibers as a scaffold and finally undergo terminal differentiation to transmit and receive information. As neurogenesis progresses, diverse subtypes of projection neurons are generated sequentially and their migration occurs in an inside-out manner: neurons generated first occupy the deepest layers of the future six layered neocortex whereas later born neurons by-pass earlier born neurons and settle in more superficial layers (Gupta et al., 2002).

Unlike the projection neurons, the inhibitory interneurons of the cerebral cortex are generated from distinct progenitors in the germinal zones of the ventral telencephalon, mainly within the medial and caudal ganglionic eminences. Interneurons then undertake tangential migration toward the cortex using different routes according to the time and place of birth. Upon arrival in the cortex, interneurons switch their mode of migration from tangential to radial and reach their destination layer based on their molecular subtype, origin and birthdate, on cortical cues and make local connections with pyramidal cells (Bartolini et al., 2013; Guo and Anton, 2014).

The development of each category of cortical neurons is thus a multistep process, which involves dramatic morphological changes at each step of the process. These changes are mediated by an extensive and dynamic remodeling of the cytoskeleton. Among the major regulators of cytoskeletal properties, the small signaling molecules of the Rho GTPase family play essential functions in cerebral cortex development. In this review, we will address the role of Rho GTPases in cortical projection neuron development, focusing on *in vivo* studies, and discuss how dysfunctional Rho GTPase signaling may contribute to cortical malformations.

## The Rho GTPase family

**Figure 1:**
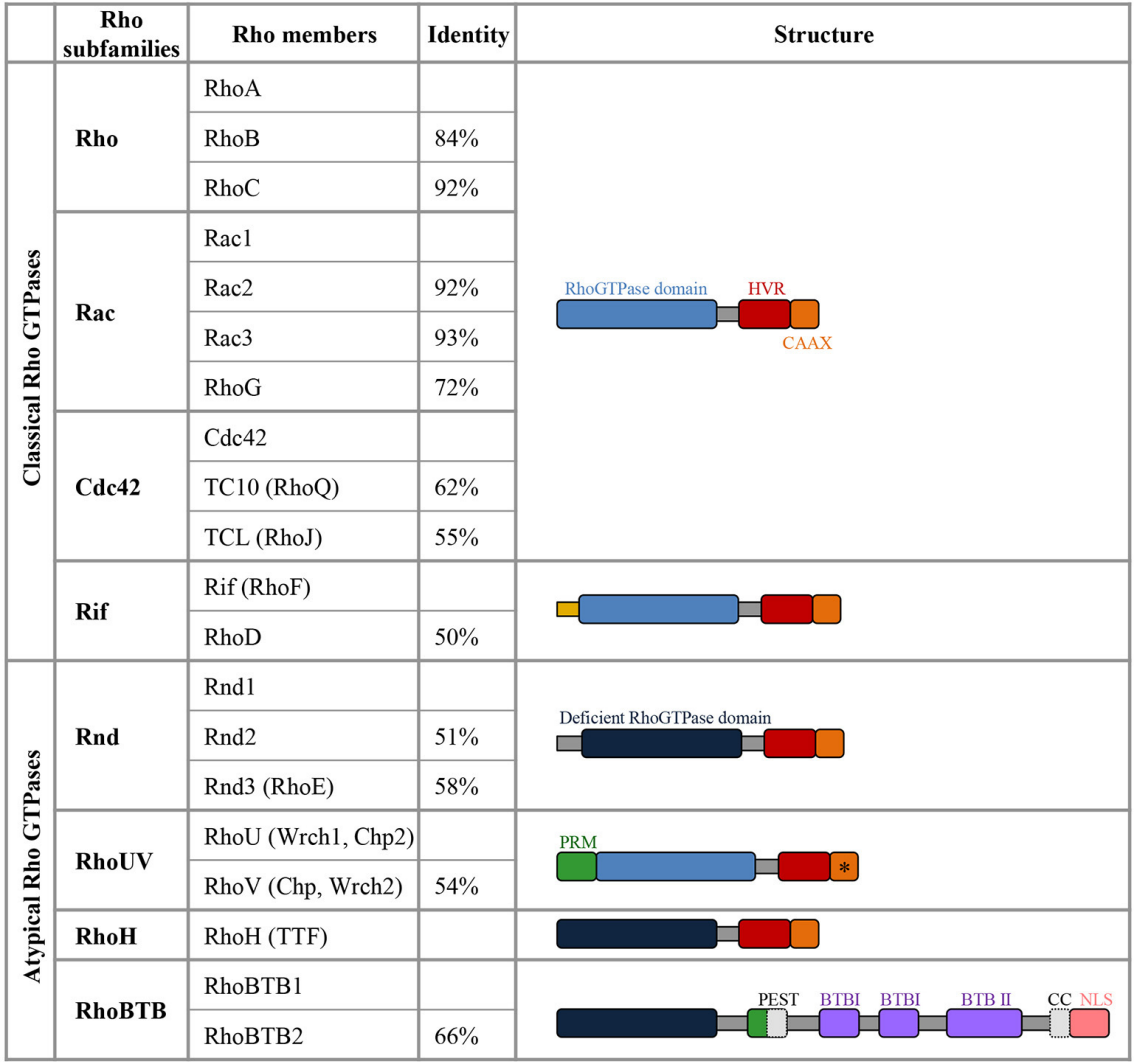
**Members of the Rho GTPase family**. The column identity indicates the percentage of amino-acid sequence identity of a specific Rho GTPase compared with the first member of the corresponding subfamily (Heasman and Ridley, 2008). Among the atypical members, Rnd1, Rnd2, Rnd3, RhoH, RhoBTB1, and RhoBTB2 lack amino acids in the GTPase domain that are critical for GTPase activity, they are thus constitutively bound to GTP and do not detectably hydrolyse GTP (deficient Rho GTPase domain in dark blue). RhoV and RhoU harbor GTPase activity but they are atypical in this family as they display a high intrinsic guanine nucleotide exchange activity and are predominantly in the GTP-loaded conformation. In the C-terminal domain, the Hyper Variable Region (HVR in red) differs not only between the Rho GTPase subclasses but also within the same subclass in terms of the presence of either a polybasic region or a palmitoylation site (Roberts et al., 2008). The polybasic region and palmitoylation site present in the HVR are involved in targeting the GTPases to plasma membrane or endomembrane compartment. The C-terminal CAAX-box (C, cysteine; A, Aliphatic Amino acid; X, any amino acid; in orange) contains a cysteine residue, which is crucial for prenylation that adds a farnesyl or geranylgeranyl group, enhancing the interaction with membranes and very often defining the localization to specific membrane compartments. The CAAX with a * indicates that RhoV does not seem to have a functional CAAX box and the CAAX motif of RhoU is apparently not undergoing prenylation (Aspenstrom et al., 2007). The Rif members have a N-terminal extension (in yellow) that is unique to this subgroup. RhoUV proteins display a proline rich motif (PRM, in green), which is also present in RhoBTB1 and RhoBTB2. RhoBTB proteins contain two Broad complex/Tramtrack/Bric-a-brac domains (BTB). RhoBTB family members harbor different domains involved in protein-protein interaction (in gray: Coiled Coil, CC, only present in RhoBTB1 and PEST domain only present in RhoBTB2) and they display a nuclear localization signal (NLS, in pink).

The Rho family of GTPases represents a subgroup of the Ras superfamily of small GTP binding proteins (Heasman and Ridley, 2008). The most extensively studied members of the Rho family are RhoA (Ras homologous member A), Rac1 (ras related C3 botulinum toxin substrate 1) and Cdc42 (cell division cycle 42) but this family actually includes 20 members which are subdivided into 8 subgroups based on amino-acid sequence similarities (Figure 1).

**Figure 2:**
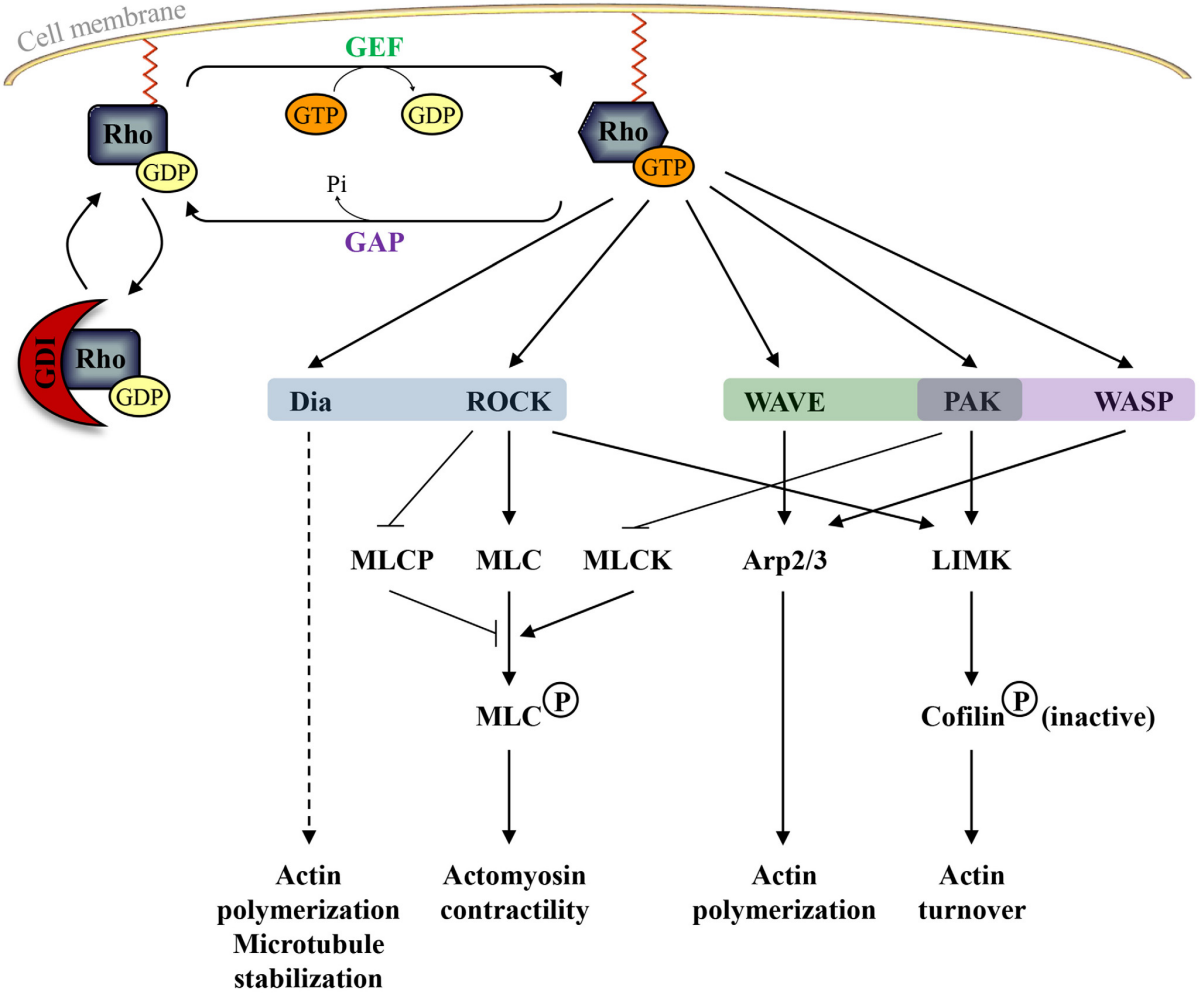
**The classical Rho GTPase cycle and the main pathways regulated by active RhoA (in blue), Rac1 (in green), and Cdc42 (in purple)**. Guanine nucleotide-exchange factors (GEFs) activate Rho GTPases by promoting the release of GDP and the binding of GTP whereas GTPase-activating proteins (GAPs) inactivate Rho GTPases by increasing the intrinsic GTPase activity of Rho proteins. Guanine nucleotide-dissociation inhibitors (GDIs) sequester RhoGTPase in their inactive state and protect them from degradation. In their active form, Rho GTPases can bind to different effector molecules. Dia: Diaphanous-related formins; ROCK: Rho Kinase; MLCP: myosin light chain phosphatase; MLC: myosin light chain; MLCK: myosin light chain kinase; WAVE: Wiskott–Aldrich syndrome protein family verprolin homolog; Arp2/3: actin-related proteins 2 and 3; PAK: p21-activated kinases; LIMK: Lin-11, Isl-1, and Mec-3 kinase; WASP: Wiskott-Aldrich syndrome protein.

Like other small GTP-binding proteins of the Ras superfamily, most Rho GTPases cycle between GTP (active) and GDP (inactive)—bound states. The GDP/GTP cycle is promoted by the activity of two classes of molecules, guanine nucleotide exchanging factors (GEFs) and GTPase activating proteins (GAPs). GEFs facilitate the exchange of GDP with GTP, resulting in protein activation. GAPs instead stimulate the intrinsic enzymatic activity of the GTPases, which promotes hydrolysis of GTP into GDP. GAP activity therefore ends the cycle and returns the GTPases in their inactive state (Bos et al., 2007) (Figure 2). Over 80 GEFs and more than 70 GAPs have been reported, suggesting that Rho GTPase regulation is complex. In addition, Rho GTPases can bind to proteins known as guanine-nucleotide dissociation inhibitors (GDIs). RhoGDIs sequester RhoGTPase in their inactive state and protect them from degradation (Boulter et al., 2010) (Figure 2). When bound to GTP, Rho GTPases exhibit the correct structural conformation to interact with effectors and initiate downstream signaling to regulate actin and microtubule components of the cytoskeleton (Jaffe and Hall, 2005) (Figure 2). However, some members of the family do not follow this classical scheme of activation and are described as atypical. These atypical Rho GTPases are predominantly GTP bound, owing either to aminoacid substitutions at residues that are crucial for GTPase activity (for example in Rnd proteins) or owing to increased nucleotide exchange (for example in RhoU). Therefore, their expression, localization, stability and phosphorylation control their activity rather than the GDP/GTP switch (Aspenstrom et al., 2007) (Figure 1).

Experimental data indicating the importance of the Rho family of small GTPases in cerebral cortex development have been accumulated over the past few years. Much of our understanding on their role comes from *in vitro* studies (Govek et al., 2005). Nevertheless, in the last years, the use of conditional mutant mice and the development of techniques such as *in utero* electroporation have allowed to highlight and clarify their functions *in vivo*.

## Expression of Rho GTPases in the developing cerebral cortex

Most of these *in vivo* studies have focused on RhoA, Rac1, and Cdc42, and more recently on Rnd2 and Rnd3. The functions of the other members of the Rho GTPase family in cortical development remain largely unknown.

**Figure 3:**
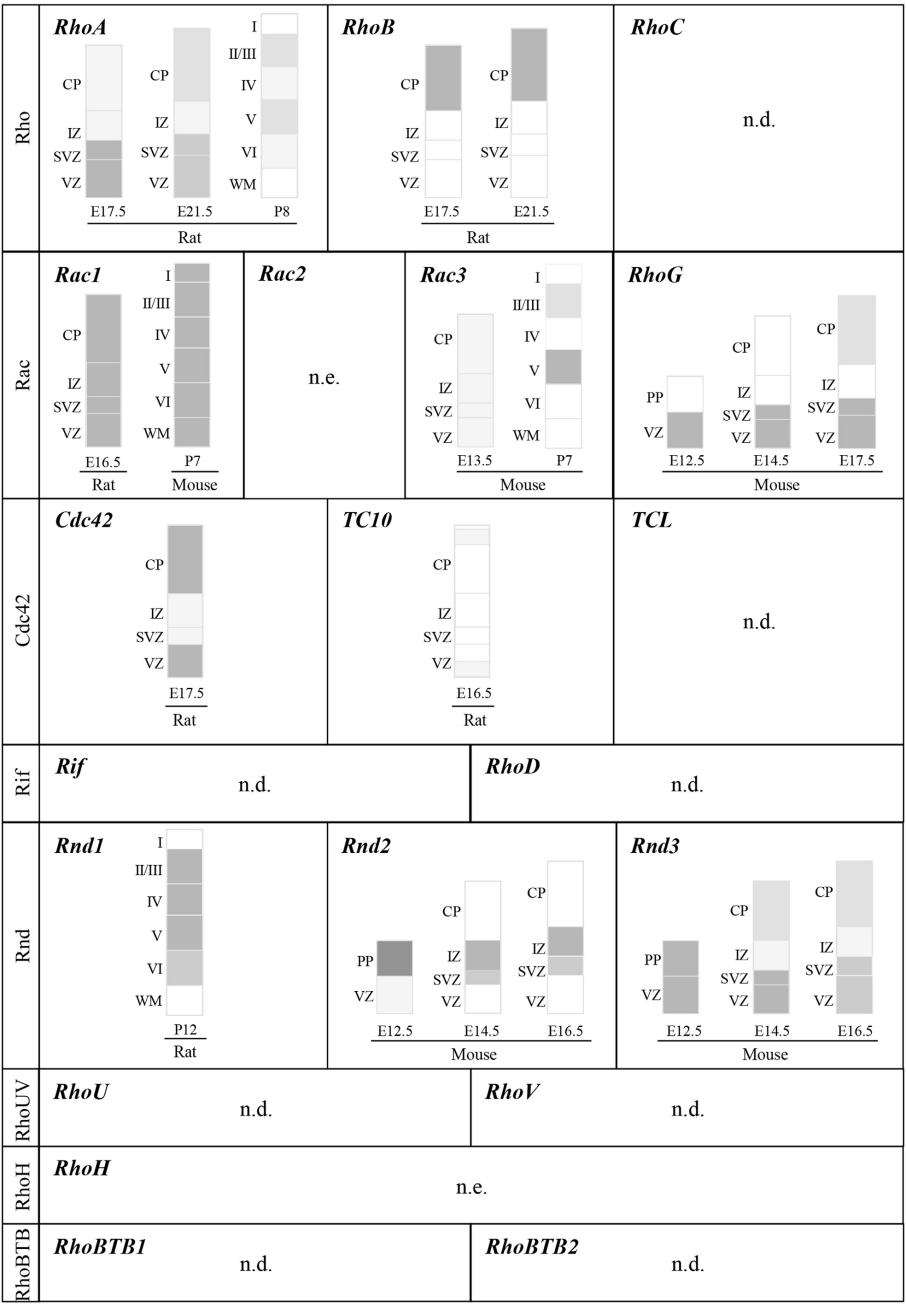
**Expression of Rho GTPase genes in the developing cerebral cortex**. Schematic representation of cortical domains depicting the expression pattern of Rho GTPase genes within the murine cerebral cortex at different developmental stages. To simplify the representation, this figure does not include spatial differences, which should be however kept in mind since all cortical areas do not develop at the same rate and timing. References cited in the Section EXPRESSION OF Rho GTPases IN THE DEVELOPING CEREBRAL CORTEX should be consulted for details on expression patterns and changes in expression during development. Dark gray and light gray indicate higher and lower relative levels of expression, respectively. VZ: ventricular zone; SVZ: ventricular zone; IZ: intermediate zone; CP: cortical plate; WM: white matter; PP: preplate; n.e.: not expressed in the brain; n.d.: expression in the cerebral cortex not determined.

In the Rho subgroup, *RhoA* and *RhoB* are highly expressed in the embryonic cerebral cortex but with distinct patterns (Olenik et al., 1999; Ge et al., 2006; Heng et al., 2008). *RhoA* mRNA is mainly expressed in domains of cellular proliferation whereas *RhoB* mRNA is absent in the proliferative zones but highly expressed in the cortical plate (CP) where neurons migrate or settle at the end of their migration (Figure 3). *RhoC* mRNA is detected in the nervous system but its distribution in the developing cerebral cortex has not been thoroughly examined (Erschbamer et al., 2005). Three of the 4 vertebrate Rac-related genes, namely *Rac1, Rac3*, and *RhoG*, are expressed in the nervous system (Figure 3) (de Curtis, 2008). Most studies on cortical neuron development have focused on Rac1, whereas only a few studies exist on Rac3 and RhoG, despite their expression in the developing cerebral cortex. However, their temporal expression is different as illustrated by low levels of *Rac3* mRNA in the embryonic cortex where *Rac1* and *RhoG* mRNA are highly expressed (Ishikawa et al., 2002; Bolis et al., 2003; Corbetta et al., 2005; Fujimoto et al., 2009). Instead, *Rac3* is mainly expressed in the postnatal cortex (P7) in layer V and to a lesser extent in layers II-III (Corbetta et al., 2005). In the Cdc42 subfamily, Cdc42 is expressed throughout the developing cerebral wall (Olenik et al., 1999; Yokota et al., 2010). Cdc42 protein is particularly enriched at the apical/ventricular side of the neuroepithelium and is present in basally located post-mitotic neurons (Cappello et al., 2006). In contrast to Cdc42, *TC10* expression is very low in the embryonic cerebral cortex (Figure 3) (Tanabe et al., 2000). Its expression in the brain however increases with development (Tanabe et al., 2000; Abe et al., 2003). Similarly, very low levels of *TCL* mRNA are detected in the brain, at least in adult murine tissue (Vignal et al., 2000). Concerning the last subfamily of classical Rho GTPases, although *RhoF* and *RhoD* are expressed in the adult brain (Murphy et al., 1996; Ellis and Mellor, 2000) and have been shown to regulate neuronal development *in vitro* (Hotulainen et al., 2009; Gad and Aspenstrom, 2010), their expression pattern in the developing cerebral cortex has not been described.

Among the atypical Rho GTPases, the expression profile of Rnd members in the developing cerebral cortex has been the most well-characterized (Figure 3). At E14.5, *Rnd1* mRNA levels are very low in the cortex but they gradually increase to peak at postnatal stages (Ishikawa et al., 2003). Conversely, the expression of *Rnd2* and *Rnd3* is high in the embryonic cerebral cortex but show different distribution in the cortical domains (Figure 3) (Azzarelli et al., 2014). *RhoV* mRNA is expressed in the human fetal brain (Katoh, 2002) and *RhoU* mRNA is found in the adult human cerebral cortex (Kirikoshi and Katoh, 2002) but their expression in the developing cerebral cortex has not been explored. Like Rac2, the expression of RhoH is restricted to hematopoietic stem cells (Troeger and Williams, 2013). Finally, the three members of the RhoBTB subfamily are expressed in the brain with *RhoBTB3* showing the highest expression levels in the adult tissue (Ramos et al., 2002). However, RhoBTB3 is not included in the Rho GTPase family since it does not seem to have a GTP-binding domain, at least it does not contain a consensus GTP-binding motif (Aspenstrom et al., 2007).

## Rho GTPases and regulation of cortical projection neuron development

### Rho GTPases and regulation of adherens junction integrity

**Figure 4:**
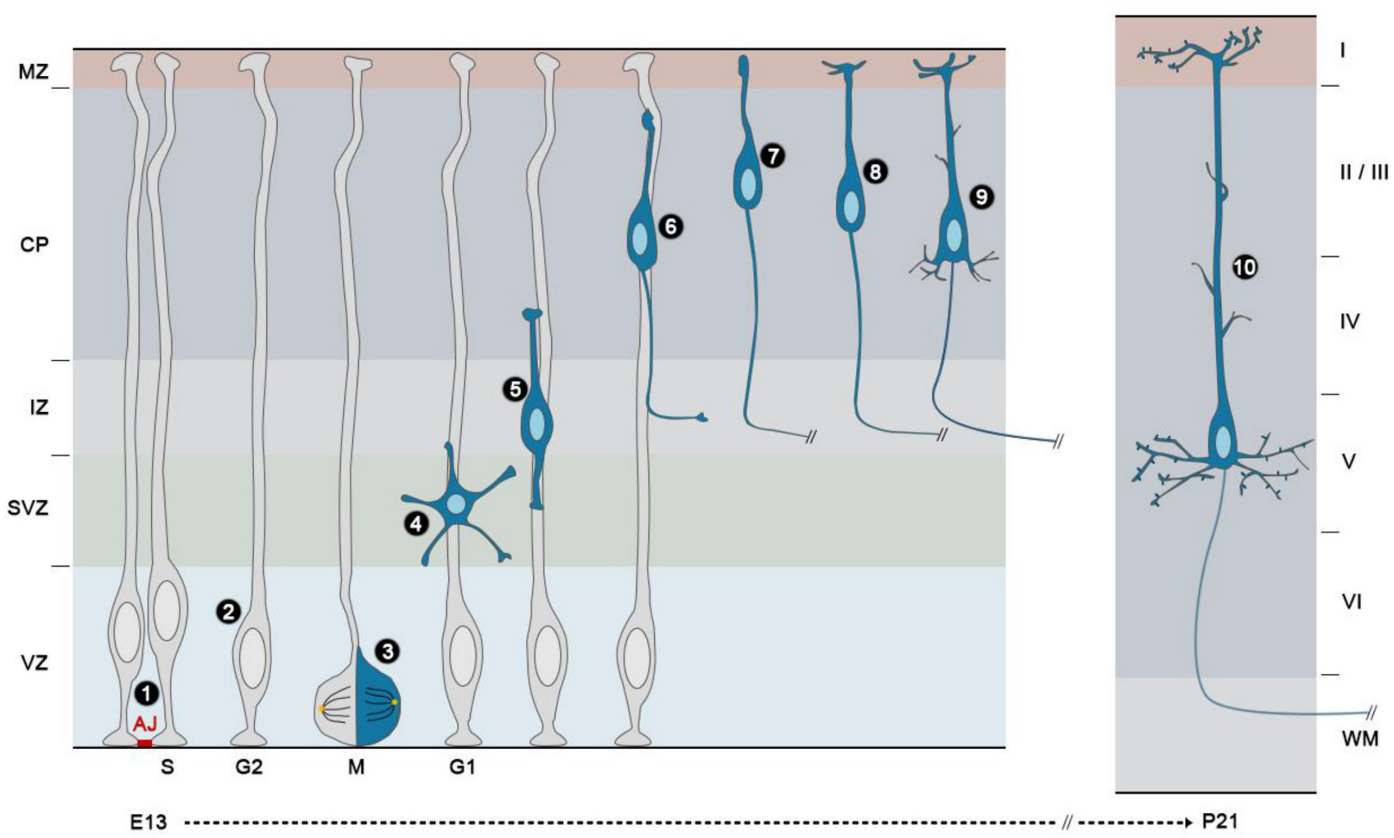
**Development of projection neurons in the mouse cerebral cortex**. The neural stem/progenitor cells of the cerebral cortex or radial glial cells (RGs) are highly polarized cells that are attached to one another in the ventricular zone (VZ) by apically located adherens junctions (AJ) ❶. Their nuclei migrate during cell cycle progression from a basal position during S phase to an apical position during mitosis (M), and the nuclei of the daughter cells migrate back to enter S phase on the basal side of the VZ, in a process called interkinetic nuclear migration (INM) ❷. During the peak of neurogenesis, most radial glial cells divide asymmetrically with a vertical cleavage plane ❸. In these divisions, one daughter remains a RG and continues to divide at the ventricular surface, whereas the other detaches from the ventricular surface, move radially away to the subventricular zone (SVZ)/lower intermediate zone (IZ) and acquires a multipolar shape ❹. Then, nascent neurons become bipolar, extending a leading process toward the pial surface and a trailing process in the opposite direction ❺. Upon multi to bipolar transition, newborn neurons establish contacts with RG fibers and subsequently use them as a scaffold to migrate to the upper part of the cortical plate (CP) using a mode of migration called locomotion ❻. During this phase the trailing process becomes the axon and extends to its final destination. Once cortical neurons reach the upper part of the CP and right after their leading process makes contact with the marginal zone (MZ), they detach from the RG fibers and execute a terminal somal translocation ❼. The leading process then gives rise to the apical dendrite, which initiates local branching in the MZ ❽. Basal dendrites subsequently appear as well as oblique side branches emerging from the apical shaft ❾. At this stage, the cell body of early-born neurons translocate ventrally as neurons born at later stages bypass their predecessors. The final step in cortical projection development is the apparition and maturation of spines. For example, in layer V pyramidal neurons, spines are morphologically mature at P21 on apical dendrites ❿.

Like NEs, RGs are highly polarized along their apico-basal axis. They are attached to the luminal surface of the ventricle on their apical side, where they form adherens junctions (AJs) with neighboring RGs (Figure 4, ❶), and to the basal lamina via integrins (Gotz and Huttner, 2005). Their cell bodies are retained within the ventricular zone (VZ), a defined region next to the ventricles. AJs between RGs maintain VZ integrity and cortical architecture as well as RG behavior by anchoring a variety of proteins (N-cadherin, β-catenin, αE-catenin) to the actin cytoskeleton (Gotz and Huttner, 2005).

RhoA plays a critical role in the maintenance of these adherens junctional complexes. Indeed, the deletion of *RhoA* by *FoxG1^Cre^* (Katayama et al., 2011) or *Emx1-Cre* mediated recombination (Cappello et al., 2012) leads to a disorganization of the VZ surface and to a loss of catenin expression at the apical surface around E14.5. In these mutants, rings of intense catenin expression are instead observed inside the brain mass (Katayama et al., 2011; Cappello et al., 2012). Similarly, perturbation of Rho by electroporation either with the RhoA/B/C inhibitor C3 transferase or with *RhoA, RhoB, RhoC* shRNAs impairs the apical actin filament belt and the apico-basal polarity of electroporated cells (Thumkeo et al., 2011). Expression of dominant-active Rho also affects the actin structure and the apical localization of N-cadherin, suggesting that balanced Rho activity is necessary for maintaining AJ integrity in RGs (Thumkeo et al., 2011) (Table 1). While Rho is essential for the maintenance, Cdc42 seems to be essential for the initial formation of apical AJs since the disappearance of apical proteins (PAR6, aPKC, E-cadherin, β-catenin, F-actin and Numb) as well as the apico-basal polarity occurs as early as E10.5 in *FoxG1^Cre^ Cdc42* null embryos (Chen et al., 2006). A similar phenotype is found after deletion of *Cdc42* by *Emx1^Cre^* or *Nestin-Cre* mediated recombinations (Cappello et al., 2006; Garvalov et al., 2007; Peng et al., 2013) (Table 1). Although Rac1 and Cdc42 share many effectors, junction formation and cell polarity during cerebral cortex development specifically require Cdc42 but not Rac1. In Rac1 mutants, VZ progenitors are not tightly packed or radially oriented as in controls. However, this phenotype is not due to an alteration of VZ progenitor polarity since the loss of *Rac1* does not affect the expression pattern of β-catenin and cadherin (Leone et al., 2010). Accordingly, the expression of phosphorylated PAK (p21- activated kinase), which is a direct downstream effector of both Rac1 and Cdc42, at the apical surface of VZ progenitors is affected by the loss of *Cdc42* but not *Rac1* (Leone et al., 2010), further demonstrating that these two Rho GTPases perform non-overlapping and non-redundant functions in the VZ. More recently, the atypical Rho GTPase Rnd3 has also been shown to maintain the integrity of the junctions between RGs through regulation of RhoA and the actin cytoskeleton (Pacary et al., 2013) (Table 1).

**Table 1:** **Regulation of cortical projection neuron development by Rho GTPases (*in vivo* studies)**.

**Rho GTPase**	**Genetic modification**	**Analysis**	**Cortical phenotype(s)**	**References**
**ADHERENS JUNCTION INTEGRITY**				
RhoA	*RhoA*cKO x *FoxG1^Cre^*	E14.5	Disorganization of VZ surface, loss of αE-catenin apical expression	Katayama et al., 2011
	*RhoA*cKO x *Emx1-Cre*	E12-E14	Loss of β-catenin, pan-cadherin, Par-3 apical expressions; disorganized arrangement of radial glia somata and processes	Cappello et al., 2012
RhoA, B, C	*C3-GFP* (E15)	E16	Loss of apico-basal polarity (round cells with abnormal processes projecting in random positions); disruption of VZ architecture, loss of apical actin filament belt and loss of apical localization of N-cadherin	Thumkeo et al., 2011
	shRNAs coelectroporation (E15)	E18	Impairment of actin belt and apico-basal polarity	Thumkeo et al., 2011
	*RhoV14-GFP* (E15)	E16	Loss of the apical process, of the actin filament belt and of apical N-cadherin expression	Thumkeo et al., 2011
Cdc42	*Cdc42*cKO x *FoxG1^Cre^*	E10.5	Loss of PAR6, aPKC, E-cadherin, β-catenin, F-actin and Numb apical expressions; loss of apico-basal polarity in neuroepithelial cells	Chen et al., 2006
	*Cdc42*cKO x *Emx1^Cre^*	E11-E14	Loss of β-catenin, F-actin, Par complex protein expressions at the apical surface; loss of the apical process	Cappello et al., 2006
	*Cdc42*cKO x *Nestin-Cre*	E18.5	Disruption of the apical surface	Garvalov et al., 2007
		E14.5	Loss of apico-basal polarity in VZ	Peng et al., 2013
Rnd3	*Rnd3* shRNA (E14.5)	E15.5	Disruption of β-catenin, N-cadherin, F-actin and ZO-1 distribution at the ventricular surface; detachment of the apical process from the ventricular surface	Pacary et al., 2013
**INTERKINETIC NUCLEAR MIGRATION**				
Cdc42	*Cdc42*cKO x *Emx1^Cre^*	E10.5	Delay of basal to apical INM (BrdU pulse)	Cappello et al., 2006
	*Cdc42*cKO x *Nestin-Cre*	E16.5	Impairment of INM (BrdU pulse)	Peng et al., 2013
Rac1	*DN-Rac1* (E13.5)	E14.5	Delay of basal to apical INM (live imaging)	Minobe et al., 2009
Rnd3	*Rnd3* shRNA (E14.5)	E15.5	Delay of basal to apical INM (BrdU pulse)	Pacary et al., 2013
**CORTICAL PROGENITOR PROLIFERATION**				
RhoA	*RhoA*cKO x *FoxG1^Cre^*	E13.5	Expansion of Pax6^+^ progenitor pool	Katayama et al., 2011
		E14.5	Ki67^+^ progenitors are intermingled with post-mitotic neurons or form rosette-like structures	
	*RhoA*cKO x *Emx1-Cre*	E14.5	↑ total number of pHH3^+^ cells in the caudal part of the cortex	Cappello et al., 2012
		E16.5	↑ total number of pHH3^+^ cells the rostral part of the cortex	
		E14.5	Aberrant location of Pax6^+^ and Tbr2^+^ cortical progenitors	
Rac1	*Rac1*cKO x *FoxG1^Cre^*	E16.5-E18.5	↓ Ki67^+^ population; acceleration of cell-cycle exit	Chen et al., 2009
	*Rac1*cKO x *FoxG1^Cre^*	E14.5	↓ Tbr2^+^ population proliferation within the SVZ; premature cell cycle exit and differentiation of Tbr2^+^ progenitors	Leone et al., 2010
RhoG	*CA-RhoG* (E14)	E16	↑ Ki67^+^ cells	Fujimoto et al., 2009
	*RhoG* shRNA (E14.5)	E16	↓ Ki67^+^ cells	
Rnd3	*Rnd3* shRNA (E14.5)	E16.5	↑ Tbr2^+^ population; ↑ pHH3^+^ and Ki67^+^ cells in the SVZ	Pacary et al., 2013
**CLEAVAGE PLANE ORIENTATION**				
Rnd3	*Rnd3* shRNA (E14.5)	E15.5	↑ fraction of radial glial cells dividing with an oblique or horizontal cleavage plane	Pacary et al., 2013
**CELL FATE**				
Cdc42	*Cdc42*cKO x *Emx1^Cre^*	E14	Apical to basal fate conversion	Cappello et al., 2006
**RADIAL MIGRATION**				
Rac1	*Rac1*cKO x *FoxG1^Cre^*	E18.5	Delay in radial migration	Chen et al., 2007
	*Rac1*cKO x *FoxG1^Cre^*	E17	Laminar disorganization; disorganization of radial glia fibers	Leone et al., 2010
	*DN-Rac1* (E14.5)	E17	Cells fail to extend a leading process	Kawauchi et al., 2003
		P0, P4	Accumulation of cells in the IZ	
	*CA-Rac1* (E15.5)	P2	Cells fail to extend a leading process	Konno et al., 2005
	*DN-Rac1* (E15.5)	P2	Accumulation of cells in the IZ	
	*CA-Rac1* or *DN-Rac1* (E14.5)	E18.5	Accumulation of cells in the IZ	Yang et al., 2012a
	*Rac1* shRNA (E14.5)	E18.5	Accumulation of cells in the IZ; defect in the formation of the proximal cytoplasmic dilation in the leading process	Yang et al., 2012a
	*WT-Rac1* (E14.5)	E18.5	Promotion of neuronal migration	Yang et al., 2012a
Rnd2	*Rnd2* shRNA (E14.5)	E17.5	↓ fraction of cells reaching the CP; multipolar cells with longer processes in the IZ	Heng et al., 2008; Pacary et al., 2011
Rnd3	*Rnd3* shRNA (E14.5)	E17.5	↓ fraction of cells reaching the CP; multiple thin processes extending from the enlarged leading process in CP; impairment of the centrosome-nucleus coupling	Pacary et al., 2011
Cdc42	*CA-Cdc42* or *DN-Cdc42* (E15.5)	P2	Inhibition of radial migration	Konno et al., 2005
RhoA	*DN-RhoA* (E14.5)	E17.5	Promotion of neuronal migration	Nguyen et al., 2006
	*RhoA* shRNA (E14.5)	E17.5	Defects in neuronal migration	Pacary et al., 2011
	*WT-RhoA* or *RhoA S26A* (higher RhoA activity) (E14.5)	E17.5	Defects in neuronal migration	Tang et al., 2014
	*RhoA*cKO x *Emx1-Cre*	E17.5	Migration defects secondary to radial glia scaffold disruption; normal migration of mutant cells in a wild-type environment	Cappello et al., 2012
	*RhoA*cKO electroporation with Cre (E14)	E17	Faster migration	Cappello et al., 2012
	*RhoA*cKO electroporation with a fast cycling RhoA mutant (E14)	E17	Delay in migration	Cappello et al., 2012
**AXON DEVELOPMENT**				
Cdc42	*Cdc42*cKO x *Nestin-Cre*	E18.5	Few, short and sparse axonal tracts	Garvalov et al., 2007
		P0	↓ or loss of axonal tracts	
	*Cdc42*cKO x *GFAP-Cre*	P14	Disruption of callosal axon growth and organization	Yokota et al., 2010
Rac1	*Rac1*cKO x *FoxG1^Cre^*	E18.5	Absence of anterior commissure, failure of corpus callosal axons to cross the midline, defasciculation or projection defects of thalamocortical and corticothalamic axons	Chen et al., 2007
	*Rac1*cKO x *Emx1^Cre^*	Adult	Impaired formation of fiber tracts in the corpus callosum and anterior commissure	Kassai et al., 2008
**DENDRITE DEVELOPMENT**				
Cdc42	pNeuroD1-*CA-Cdc42* (E15.5)	P23	↓ dendrite branching and complexity in layer II/III pyramidal neurons	Rosario et al., 2012
**CELL DEATH**				
RhoA	Mouse line expressing	P5	↓ apoptosis in the somatosensory cortex	Sanno et al., 2010
	*DN-RhoA* in neurons	P65	↑ density and absolute number of neurons in the somatosensory cortex (projection neurons)	
Rac1	*Rac1*cKO x *FoxG1^Cre^*	E14.5-E18.5	↑ active Caspase-3^+^ and TUNEL^+^ cells	Chen et al., 2007
	*Rac1*cKO x *FoxG1^Cre^*	E14.5-E17.5	↑ number of TUNEL^+^ cells at E14.5 but no difference at E17.5	Leone et al., 2010

*VZ: ventricular zone, SVZ: subventricular zone, IZ: intermediate zone, CP: cortical plate, INM: interkinetic nuclear migration, CA: constitutively active, DN: dominant-negative*.

### Rho GTPases and regulation of interkinetic nuclear migration

In addition to apico-basal polarity, another hallmark of NEs retained by RGs is interkinetic nuclear migration (INM), a process whereby nuclei change position along the apico–basal axis during the course of the cell cycle. In NEs, this INM spans the entire apical–basal axis of the cell, with the nucleus migrating to the basal side during the G1 phase of the cell cycle, staying at the basal side during S phase, migrating back to the apical side during the G2 phase and undergoing mitosis at the ventricular surface. In RGs, the same mitotic behavior occurs, except that is confined to the portion of the cell in the VZ (Figure 4, ❷). As a consequence of this movement, the neuroepithelium and the VZ appear pseudo-stratified (Taverna and Huttner, 2010). The precise role of INM during cortical neurogenesis is still an unresolved question. INM might allow packing an increasing number of progenitor cells within a limited ventricular surface or it might regulate progenitor fate by influencing the exposure of progenitor nuclei to proliferative vs. neurogenic signals (Taverna and Huttner, 2010; Spear and Erickson, 2012).

Both microtubule-based motors and actomyosin seem to participate in either direction of INM, although to a different extent depending on the system (Taverna and Huttner, 2010; Lee and Norden, 2013). In the developing cerebral cortex, a few studies have implicated the Rho GTPases in the regulation of this process. In particular Cdc42, Rac1, and Rnd3 have been shown to control the basal-to-apical movement. Indeed, this movement is delayed in *Cdc42* deficient mice (Cappello et al., 2006) or after electroporation of a dominant negative (DN) form of Rac1 (Minobe et al., 2009) (Table 1). However, it is not known whether microtubule or actomyosin networks mediate the effects of Rac1 and Cdc42. In contrast, the impairment of basal to apical INM after *Rnd3* silencing in the embryonic cerebral cortex is rescued by co-expression of a constitutively active form of cofilin (cofilin^S3A^), demonstrating that Rnd3-mediated disassembly of actin filaments coordinates the cellular behavior of RGs during INM at least during the apical nuclear movement (Pacary et al., 2013).

### Rho GTPases and regulation of progenitor cell division, proliferation and cell fate

At early stages of corticogenesis, NEs divide symmetrically to self-renew and expand their pool. Following the transition to the RG fate, some progenitor cells begin to divide asymmetrically to generate neurons directly or indirectly through the production of IPs or oRGs (Laguesse et al., 2014).

After their generation by asymmetric division of RGs, IPs, also called basal progenitors, retract their apical and basal processes, exhibit a multipolar morphology and migrate basally (Figure 4, ❸ and ❹) before they undergo mitosis. This second pool of proliferative progenitors undergo one or more symmetric cell divisions (Noctor et al., 2004), which significantly increases the yield of cortical neurons derived from a single RG. The accumulation of these dividing progenitors in basal regions starts around E13 and it determines the formation of the subventricular zone (SVZ). Whereas NEs and RGs express identical markers like the transcription factor Pax6 (Gotz et al., 1998), IPs are identified by the absence of Pax6 and by the expression of the transcription factor Tbr2 (Englund et al., 2005).

oRGs, also known as basal radial glia cells, were first discovered in human and ferret brains (Fietz et al., 2010; Hansen et al., 2010), and were initially proposed to be a specific feature of gyrencephalic brains. However, they have also been identified in the rodent brain, where they account for less than 10% of total cortical progenitors vs. 40% in human (Hansen et al., 2010; Wang et al., 2011), and in lissencephalic primate brains (Garcia-Moreno et al., 2012; Kelava et al., 2012). oRGs arise from the division of RGs as they delaminate from the apical surface and translocate their nuclei in the outer portion of the SVZ, where they start dividing. To note, this translocation of the soma along the basal fiber toward the CP, a process termed mitotic somal translocation, requires activation of the Rho effector ROCK (Ostrem et al., 2014). In contrast to IPs, oRGs maintain molecular characteristics of RGs such as expression of Pax6 and can divide either symmetrically to expand their number or asymmetrically to self-renew and give birth to new neurons (Hansen et al., 2010; Reillo et al., 2011).

The balance between proliferation and differentiation of these different categories of progenitors is tightly regulated and is fundamental for the generation of appropriate number of cortical neurons. Among the Rho GTPases, RhoA, Rac1, RhoG, and Rnd3 have a crucial role in the control of this balance. Indeed, conditional deletion of *RhoA* in cortical progenitors using *FoxG1^Cre^* mice causes hyperproliferation, which results in the expansion of the progenitor pool and exencephaly-like protrusions (Katayama et al., 2011). Similarly, the loss of *RhoA* by *Emx1-Cre* mediated recombination increases proliferation in a region-specific manner within the cerebral cortex, starting at occipital regions at E14 and later at E16 in rostral parts, and this phenotype is associated with an aberrant location of Pax6+ and Tbr2+ progenitors (Cappello et al., 2012) (Table 1). In contrast to RhoA, the forebrain-specific loss of *Rac1* by *FoxG1^Cre^* leads to a SVZ-specific reduction in proliferation, a concomitant increase in cell cycle exit and premature differentiation (Chen et al., 2009; Leone et al., 2010) (Table 1). How RhoA and Rac1 differently affect proliferation of cortical progenitors is not known. However, studies in other cellular systems have shown that RhoA and Rac1 influence the levels of cyclins during G1 progression. Interestingly, Rac1, but not Rho, stimulate cyclinD1 transcription when ectopically expressed in cells (Jaffe and Hall, 2005). In addition, RhoA might regulate proliferation of cortical progenitors during cytokinesis through its action on F-actin and myosin II into the actomyosin contractile ring (Marzesco et al., 2009) or through its action on actomyosin filaments at the cell cortex which influence mitotic spindles and the plane of cell division (see next paragraph) (Jaffe and Hall, 2005). Rnd3 has also been shown to control specifically the proliferation of basal progenitors via cyclinD1 but in an opposite manner, i.e., *Rnd3* silencing increases SVZ proliferation (Pacary et al., 2013). Finally, RhoG, another Rac-related Rho GTPase expressed in the VZ/SVZ (Figure 3), also promotes neural progenitor cell proliferation in the mouse cerebral cortex (Table 1) through phosphatidylinositol 3-kinase (PI3K) signaling (Fujimoto et al., 2009).

In the developing cerebral cortex, cleavage plane orientation remains predominantly vertical (planar division) during the period of symmetrical division prior to neurogenesis, and throughout the period of asymmetrical division during neurogenesis (Figure 4, ❸) (Morin and Bellaiche, 2011). In these asymmetric divisions, one daughter remains a RG and continues to divide at the ventricular surface, whereas the other loses its apical attachment and becomes an IP. Fewer RGs undergo oblique or horizontal divisions, and these divisions have been proposed to generate oRGs (Morin and Bellaiche, 2011; Shitamukai and Matsuzaki, 2012).

In the mouse cerebral cortex, Rnd3 is required to maintain the vertical orientation of the cleavage plane during RG divisions. Indeed, when *Rnd3* is knockdown the fraction of RGs dividing with an oblique or horizontal cleavage plane is increased (Pacary et al., 2013). Interestingly *Rnd3*-silenced cells prematurely leave the VZ, enter the SVZ while transiently maintaining their radial glial molecular phenotype, thus showing similarities with oRGs (Pacary et al., 2013). In addition, co-electroporation of cofilin^S3A^ restores vertical cleavage-plane orientation in *Rnd3*-silenced progenitors, thus indicating that Rnd3 maintains the vertical orientation of apical divisions by remodeling the actin cytoskeleton (Pacary et al., 2013). RhoA might be also important to determine the orientation of cortical progenitor divisions as suggested by a study in the chick neuroepithelium, in which the expression of a DN form of RhoA results in random spindle orientation (Roszko et al., 2006). Inversely, deletion of *Cdc42* does not influence spindle orientation in the developing cerebral cortex (Cappello et al., 2006). However, as mentioned previously, the loss of *Cdc42* causes defects in apical process maintenance, thereby leading to an increased number of progenitors dividing at basal rather than apical positions. These progenitors convert to an SVZ fate as shown by the increase of Tbr2+ progenitors and a decrease of Pax6+ population in the mutants, which ultimately leads to a higher rate of neuron generation (Cappello et al., 2006).

### Rho GTPases and regulation of radial migration

After detachment from the ventricular surface in the VZ, nascent neurons move radially away to the SVZ/lower intermediate zone (IZ), where they acquire a multipolar shape (Figure 4, ❹). During this phase, multipolar neurons actively extend and retract dynamic processes and tend to migrate tangentially in an apparent random fashion (Noctor et al., 2004; Jossin and Cooper, 2011). Then, neurons become bipolar, extending a leading process toward the pial surface and a trailing process in the opposite direction (nascent axon) (Figure 4, ❺). Upon multi to bipolar transition, neurons establish dynamic contacts with RG fibers and subsequently use them as a scaffold to migrate to the upper part of the CP using a mode of migration called locomotion (Figure 4, ❻). This movement is characterized by repetitive cycles of synchronized steps. First, a cytoplasmic dilation forms in the proximal region of the leading process. Second, the centrosome moves toward the swelling and finally the nucleus translocates toward the centrosome, a process known as nucleokinesis. This migration cycle then starts again confering a saltatory advancement to the locomoting neurons. Finally, once cortical neurons have reached the uppermost area of the CP and right after their leading process makes contact with the MZ, they detach from the RG fibers and execute a terminal somal translocation to settle in their appropriate final position (Figure 4, ❼) (Nadarajah et al., 2001). Rac1, Rnd2, and Rnd3 are three Rho GTPases with specific functions in the control of the migratory process in the developing cerebral cortex: Rac1 signaling regulates leading process formation (Kawauchi et al., 2003; Konno et al., 2005), Rnd2 is critical for the multi to bipolar transition (Heng et al., 2008; Pacary et al., 2011) and Rnd3 is important for nuclear-centrosome coupling during locomotion (Pacary et al., 2011).

Conditional knockout of *Rac1* in the forebrain, *in utero* electroporation of DN or constitutively active (CA) forms of Rac1 as well as *Rac1* shRNA or wild-type Rac1 have demonstrated a requirement for this Rho GTPase in radial migration (Kawauchi et al., 2003; Konno et al., 2005; Chen et al., 2007; Kassai et al., 2008; Yang et al., 2012a). *Rac1* deletion using the *FoxG1^Cre^* (Chen et al., 2007) or *Emx1^Cre^* line (Kassai et al., 2008) disturbs radial migration, but the defects observed in these mice are less severe than those observed after electroporation of the DN form of Rac1 (N17-Rac1). While the migration of nascent neurons seems to be only delayed in the mutants, the inhibition of Rac1 activity with N17-Rac1 leads to an accumulation of electroporated cells in the IZ (Kawauchi et al., 2003; Konno et al., 2005; Yang et al., 2012a). In addition, N17-Rac1 expressing cells in this cortical domain fail to extend a leading process and are instead round, with short and irregular processes (Kawauchi et al., 2003). Interestingly, the electroporation of *CA-Rac1* (*V12Rac1*) induces a similar phenotype (Konno et al., 2005; Yang et al., 2012a) indicating that cycles of Rac1 activation and inactivation and thus a fine regulation of Rac1 activity is important for proper morphological polarization and migration. The difference obtained between conditional knockout mice and the electroporation of dominant mutant might reflect the ability of this mutant as well as of CA mutant to interfere with the activity of other Rho GTPases, possibly through the competitive binding with regulators like RhoGDIs (Boulter et al., 2010). Nevertheless, the requirement of Rac1 for proper radial migration has been further confirmed recently with *Rac1* shRNA (Yang et al., 2012a). Indeed, the silencing of *Rac1* blocks radial migration and disrupts the formation of the proximal cytoplasmic dilation in the leading process of migratory cortical neurons (Yang et al., 2012a). Interestingly, in this study, the authors also show that electroporation of wild-type *Rac1* promotes neuronal migration and that POSH, a Rac1-interacting scaffold protein, recruits activated Rac1 to the plasma membrane. At this site, activated Rac1 regulates actin remodeling and controls the dilation of the leading process, two key events that promote centrosomal movement and soma translocation (Yang et al., 2012a). To note, another report provides the evidence that migration defects caused by loss of *Rac1* in *Foxg1^Cre^* mice may be due, at least in part, to defects in radial glial organization (Leone et al., 2010).

*In vivo* loss of function studies on the atypical Rho GTPase Rnd3 revealed that its knockdown in migrating neurons results in enlarged leading processes with numerous branches and increases centrosome-nucleus distance in the CP, indicative of disrupted nuclear-centrosome coupling during locomotion (Pacary et al., 2011). In contrast, *Rnd2*-deficient neurons fail to leave the IZ and display long processes at the multipolar stage, suggesting that Rnd2 is critical for the multipolar to bipolar transition that occurs in the IZ (Heng et al., 2008; Pacary et al., 2011). In addition, the two proteins fail to compensate for each other during neuronal migration, further indicating that they play distinct roles in this process (Pacary et al., 2011). Despite these different functions, both Rnd2 and Rnd3 regulate neuronal migration by inhibiting RhoA. Indeed, FRET analysis *in vivo* showed that *Rnd2* or *Rnd3* silencing increases RhoA activity in cortical cells and *RhoA* knockdown rescues the migratory defects associated with *Rnd2* or *Rnd3* loss of function. The inhibitory effect of Rnd3 on RhoA activity depends on its interactions with p190RhoGAP, whereas Rnd2's RhoA inhibitory activity does not. Further, although both Rnd2 and Rnd3 regulate actin dynamics in migrating neurons, only Rnd3 promotes neuronal migration by inhibiting RhoA-mediated actin polymerization and remodeling (Pacary et al., 2011). Interestingly, the distinct subcellular localization of Rnd2 and Rnd3 and the resultant modulation of RhoA activity in different cell compartments underlie the difference in their effects. Rnd3 owes its distinct role in neuronal migration to its localization and interaction with RhoA at the plasma membrane. Rnd2 is expressed in early endosomes and can replace Rnd3 in migrating neurons if it is targeted to the plasma membrane by replacement of its carboxyl-terminal domain with that of Rnd3 (Pacary et al., 2011). However, the mechanisms by which Rnd2 promotes neuronal migration and inhibits RhoA remains unknown. The finding that Rnd3 and Rnd2 control different phases of radial migration by inhibiting RhoA in different cell compartments suggests that in cortical neurons, RhoA acts dynamically in different cellular domains to control different aspects of the migratory process.

The functions of the other most studied members of the Rho GTPase family, RhoA and Cdc42, in the control of radial migration are less well-understood. When *DN-Cdc42* (*N17Cdc42*) or *CA-Cdc42* (*V12Cdc42*) are electroporated *in utero*, radial migration is inhibited but this effect is not as strong as that seen with *DN-Rac1* or *CA-Rac1* (Konno et al., 2005). In addition, the role played by Cdc42 in migrating neurons might be different from that of Rac1 since the former is mainly localized to the perinuclear region on the side of the leading process whereas the latter is expressed at the plasma membrane (Konno et al., 2005). Similarly, strict regulation of RhoA levels and activity appear to be required for radial migration *in vivo* (Nguyen et al., 2006; Pacary et al., 2011; Cappello et al., 2012; Azzarelli et al., 2014; Tang et al., 2014). The general view proposes that RhoA activity must be down-regulated to promote radial migration of pyramidal neurons. However, the analysis of *RhoA* knockout using *Emx1-Cre* suggests that RhoA is dispensable for radial migration. In this mutant, the deletion of *RhoA* generates migration defects that are only secondary to radial glia scaffold disruption. Indeed, when *RhoA* knockout cells are transplanted in a wild type environment, they migrate normally, suggesting that there is no cell-autonomous requirement for RhoA activity during radial migration (Cappello et al., 2012). How to reconcile the data showing the requirement of RhoA for radial migration with the fact that *RhoA*-depleted neurons normally migrate in a wild type environment is still an open question. One possibility is that compensatory mechanisms might occur to replace RhoA function in *RhoA* conditional knockout cortices. Indeed, it has been shown that the related GTPase RhoB is strongly up-regulated in the absence of RhoA (Ho et al., 2008). RhoB and RhoC might substitute for RhoA activity during cortical neuron migration. These compensatory mechanisms may be in operation only when *RhoA* expression is completely abrogated by knockout deletion.

### Rho GTPases and regulation of neuronal polarization

Cortical neurons exist in a number of different shapes and sizes, although a mature neuron typically has several dendrites that receive inputs from presynaptic neurons and one axon that relays information to post-synaptic neurons. The formation of axon-dendrite polarity is thus crucial for a neuron to establish the precise information flow within the brain. Axons and dendrites differ in morphology, function, and protein and organelle composition. Although the development of these processes has been studied extensively *in vitro* using cultured embryonic hippocampal neurons, the corresponding developmental processes *in vivo* are still unclear. During corticogenesis, early electron microscopic studies revealed that projection neurons initiate their axons during migration whereas significant dendrite growth occurs after the cells have reached their final position (Shoukimas and Hinds, 1978).

#### Rho GTPases and regulation of axon formation, growth, guidance and branching

Time-lapse imaging revealed that multipolar cells in the IZ, after extending and retracting their short processes for several hours, suddenly elongate a long process tangentially. These cells then transformed into a bipolar shape, extending a pia-directed leading process (future apical dendrite), and migrate radially leaving the tangential process behind, forming an “L-shaped” axon (Figure 4, ❻) (Hatanaka and Yamauchi, 2013). Thus, during migration, the trailing process becomes the axon and extends while being guided to its final destination. Interestingly, a recent study has shown that the interaction between multipolar cells and the preexisting axons of early-born neurons is critical for axon specification. Indeed, once one of the neurites of a multipolar cell contacts the pioneering axons from the early-born cortical neurons, this neurite is stabilized, becomes the axon and extends rapidly (Namba et al., 2014). The duration of axon elongation is however variable according to the targeted area, which is more or less distant according to the final layer position of the cortical neuron (Molyneaux et al., 2007). For example, axons of corticofugal neurons in layer V reach the spinal cord around postnatal day P7 in mouse. Finally, upon reaching its target area, extensive axonal branching occurs during the formation of presynaptic contacts with specific post-synaptic partners (during the second and third postnatal week in the mouse cortex) (Lewis et al., 2013).

The regulation of axon development by Rho GTPases has been mainly studied *in vitro* and reviewed elsewhere (Govek et al., 2005; Hall and Lalli, 2010). However, their specific roles *in vivo* are less well-understood. Cdc42 seems however to be clearly required during cerebral cortex development for the efficient establishment of axonal polarity and growth. Indeed, cortices of *Cdc42* conditional knockout mice crossed with *Nestin-Cre* mice exhibit a widespread reduction of axonal tracts (Garvalov et al., 2007). This phenotype is accompanied by a specific increase in the phosphorylation (inactivation) of the Cdc42 effector cofilin (Garvalov et al., 2007). The axonal defects in *Cdc42* knockouts might be due to the increased levels of inactive cofilin since the deletion of this actin depolymerizing protein results in polarity defects analogous to the ones seen after *Cdc42* ablation (Flynn et al., 2012). In agreement with these data, the growth and organization of callosal axonal fiber tracts are also disrupted in *Cdc42* deficient mice obtained after mating *Cdc42* floxed mice with *hGFAP-Cre* line (Yokota et al., 2010). In addition to Cdc42, a role for Rnd2 in cortical axon extension has been suggested in a study showing that COUP-TFI, a transcription factor crucial for corticogenesis and arealization, promotes callosal axon elongation by finely regulating *Rnd2* expression levels (Alfano et al., 2011).

In contrast to Cdc42, the loss of *Rac1*, using *Foxg1–Cre* mice, does not prevent axonal outgrowth in cortical neurons (Chen et al., 2007). However, in these mutants, the anterior commissure is absent, and the axons of the corpus callosum and the hippocampal commissure fail to cross the midline. A similar phenotype is observed in *Rac1/Emx1^Cre^* knockout mutants (Kassai et al., 2008), demonstrating that Rac1 controls axon guidance rather than neuritogenesis. In addition, the thalamocortical and corticothalamic axons show defasciculation or projection defects in *Rac1/FoxG1–Cre* mutants (Chen et al., 2007), whereas corticospinal and corticothalamic projections are not affected in *Rac1/Emx1^Cre^* mice (Kassai et al., 2008). This phenotypic discrepancy might be due to the different pattern of Cre expression in these two Rac1 mutants. Indeed, in *FoxG1–Cre* mice, the recombinase is expressed in other regions than the telencephalon such as the thalamus (Hebert and McConnell, 2000) which might affect the development of thalamocortical projections. To note, Rac3, the other member of the Rac subfamily expressed in the brain, does not seem to have redundant functions with Rac1 since *Rac3* knockouts do not show any obvious developmental defects in the cortex (Corbetta et al., 2005).

The *in vivo* role of RhoA in cortical axon development has not been thoroughly examined but the cortical axons in *RhoA/Emx1–Cre* knockout mutants show correct morphology and projections (Cappello et al., 2012). Nevertheless, RhoA might act as a mediator for activity-dependent branch formation as suggested by a study performed in cortical explants (Ohnami et al., 2008).

#### RhoGTPases and dendrite/spine formation

In contrast to the axon, dendrites in cortical neurons form after migration ends. These structures are highly branched and this feature gives them the appearance of a tree. Initially, all excitatory cortical neurons exhibit the common shape of “pyramid,” which is characterized by a prominent apical dendrite. This first dendrite derives from the leading process and branches out in an apical tuft that terminates in layer I (Figure 4, ❽). With time, basal dendrites appear as well as oblique side branches emerging from the apical shaft (Figure 4, ❾) (Whitford et al., 2002). The dendritic trees and consequently the overall neuronal shapes vary greatly within the cortex, with neurons of layers II, III, V, and VI acquiring a pyramidal morphology, whereas those of layer IV predominantly having non-pyramidal morphologies. Therefore, even if the initial stages of dendrite formation are very similar, further maturation determines the final neuronal morphology. For example, spiny stellate neurons in layer IV start out with a pyramidal morphology, but then acquire a stellate morphology, by retracting their apical dendrite at an early postnatal age (Vercelli et al., 1992).

The final step in the acquisition of a mature dendritic morphology is the development of spines (Figure 4, ❿). These small dendritic protrusions, which contain receptors and other proteins necessary for synaptic transmission, begin to appear in the first postnatal week in mice, when the arborization of the apical and basal dendrites becomes more complex. When pyramidal neurons reach their mature morphology, they have a highly complex dendritic arbor and are covered with spines. The timing of spine development is variable among neurons that occupy different layers or cortical areas (Huttenlocher and Dabholkar, 1997; Whitford et al., 2002).

The role of Rho GTPases in dendrite and spine formation has been mainly addressed in culture. By manipulating Rho, Rac1, and Cdc42 activities in a number of experimental systems, it has become clear that each of these Rho GTPases plays a prominent role in the development of dendrite structure and that interplay between them determines the complexity of the dendritic tree (Newey et al., 2005). Studies are generally consistent with a key role for RhoA in controlling dendritic length and for Rac and Cdc42 in regulating dendrite branching and remodeling. More precisely, RhoA activation has a negative effect on dendritic arbor growth, whereas Rac1 and Cdc42 activation promote this process. Similarly, RhoA inhibits, whereas Rac1 and Cdc42 promote spine formation and maintenance (Newey et al., 2005). However, considering the large body of *in vitro* work, it is surprising how little is known about their roles *in vivo* in the context of cortical development. To our knowledge, only one study has addressed this role directly *in vivo*. Rosario and colleagues have shown that *in utero* electroporation of a CA form of Cdc42 under the control of the post-mitotic promoter NeuroD1 decreases dendrite branching and complexity in layer II/III pyramidal neurons at postnatal stages (Rosario et al., 2012). The other evidences are indirect and come from *in vivo* studies showing involvement of Rho GTPase regulators, namely GAPs and GEFs, in this process. These regulators include GAPs, such as NOMA-GAP, srGAP2 (also called FNBP2), Myo9b and RICS, as well as GEFs like kalirin (see Section Upstream Regulators of Rho GTPases and Cortical Projection Neuron Development and Table 2).

**Table 2:** **Regulation of cortical projection neuron development by GAPs and GEFs (*in vivo* studies)**.

**Genetic modification**	**Analysis**	**Cortical phenotype(s)**	**References**
**Rho GAPs**			
**Myo9b**: Myo9b regulates dendrite development			
*Myo9b* miRNA (E15.5)	P3	↓ dendrite length and number in layer II/III pyramidal neurons	Long et al., 2013
**p190-A Rho GAP**: p190RhoGAP regulates cortical axon development			
*p190-A RhoGAP* KO	E17.5	Agenesis of the corpus callosum (failure of callosal axons to cross the midline), absence of anterior and hippocampal commissures	Brouns et al., 2000
	E16.5	Defects in axon outgrowth, guidance and fasciculation	Brouns et al., 2001
**p190-B Rho GAP**: p190-B Rho GAP regulates cortical axon development			
*p190-B RhoGAP* KO	E18.5	Deficits in the formation of the corpus callosum and anterior commissure; deficits in neuronal differentiation	Matheson et al., 2006
**RICS**: RICS regulates dendrite development			
*RICS* miRNA (E15.5)	P3	↓ dendrite length and number in layer II/III pyramidal neurons	Long et al., 2013
**Rac GAPs**			
**α 2-Chimaerin**: α2-Chimaerin regulates corticospinal axon guidance			
*α2-Chimaerin* KO	Adult	Inappropriate midline crossing of corticospinal axons in the spinal cord	Beg et al., 2007; Wegmeyer et al., 2007
**srGAP2**: srGAP2 regulates radial migration, promotes spine maturation and limits spine density			
*srGAP2* shRNA (E14.5)	E18.5	Acceleration of radial migration, ↓ leading process branching	Charrier et al., 2012
*srGAP2* KO x Thy1-YFP	P18-P21	↓ width of spine heads, ↑ length of spine necks in apical oblique dendrites of layer V pyramidal neurons; ↑ density of dendritic spines	Charrier et al., 2012
**Cdc42 GAPs**			
**NOMA-GAP**: NOMA-GAP regulates dendrite complexity			
*NOMA-GAP* KO	E18.5, P5	Weak cortical MAP2 staining particularly in upper layers	Rosario et al., 2012
	Adult	↓ dendrite complexity in layer II/III pyramidal neurons (no modification in layer V)	
**Rho GEFs**			
**Lfc** (RhoA GEF): Lfc regulates cortical progenitor proliferation, neuronal production and mitotic spindle orientation			
*Lfc* shRNA (E13.5)	E15.5	↑ Ki67^+^, ↑ Pax6^+^ and ↓ Tbr2^+^ electroporated cells	Gauthier-Fisher et al., 2009
	E15.5–24 h after BrdU injection	↑ BrdU^+^ Ki67^+^ electroporated cells	
	E16	↑ apical cell divisions with a vertical plane of division	
	E17.5, P3	↓ HuD^+^ and ↓ Tuj1^+^ electroporated cells	
**Rac/Cdc42 GEFs**			
**DOCK7** (Rac/Cdc42 GEF): DOCK7 regulates cortical progenitor proliferation, neuronal production and INM			
*DOCK7* shRNA (E13.5)	E15.5–2 h after BrdU injection	↑ BrdU^+^ and ↑ pHH3^+^ electroporated cells in the VZ; ↑ BrdU^+^ nuclei at the apical surface	Yang et al., 2012a
	E15.5–15 min, 2, 4 or 6 h after BrdU injection	Acceleration of basal to apical INM	
	E15.5–24 h after BrdU injection	↑ BrdU^+^ Ki67^+^ electroporated cells	
	E15.5	↑ PAX6^+^ and ↓ Tbr2^+^ electroporated cells	
	P1.5	↓ Tuj1^+^ electroporated cells	
*DOCK7* overexpression (E13.5)	E15.5–2 h after BrdU injection	↓ BrdU^+^ and pHH3^+^ electroporated cells in the VZ; ↑ BrdU^+^ nuclei on the basal side of VZ	
	E15.5–15 min, 2, 4 or 6 h after BrdU injection	Delay of basal to apical INM; ↑ BrdU^+^pHH3^+^ cells at basal positions	
	E15.5–24 h after BrdU injection	↑ BrdU^+^ Ki67^+^ electroporated cells	
	E15.5	↑ PAX6^+^ and ↓ Tbr2^+^ electroporated cells	
	P1.5	↓ Tuj1^+^ electroporated cells	
**Kalirin** (Rac GEF): Kalirin regulates dendrite complexity and spine stability			
*Kalirin* KO	Adult (12 week old)	↓ spine density on oblique dendrites in layer V pyramidal neurons of the frontal cortex (no modification at 3 week old)	Cahill et al., 2009
	Adult (3 month old)	↓ dendrite branching and complexity in layer V pyramidal neurons	Xie et al., 2010
**P-Rex1** (Rac GEF): P-Rex1 regulates radial migration			
*DN-like P-Rex1*(E14)	P0	Accumulation of electroporated cells in the IZ	Yoshizawa et al., 2005
**Tiam1** (Rac GEF): Tiam1 regulates radial migration			
*DN-Tiam1* (E14.5)	P0, P4	Accumulation of electroporated cells in the IZ	Kawauchi et al., 2003

### Rho GTPases and regulation of cortical neuron death/survival

Programmed neuronal cell death, or apoptosis, is essential for proper cerebral cortex development, resulting in the refinement of nascent neuronal innervation and network formation (Nikolic et al., 2013). In the mouse, neuronal apoptosis takes place in the first 30 postnatal days, with a peak at P5, mostly pronounced in cortical layers II–IV. This wave of apoptosis accounts for a loss of approximately 30% of neuronal content in the cerebral cortex from birth to adulthood (Heumann et al., 1978; Heumann and Leuba, 1983).

Among the Rho GTPases, RhoA is of particular interest with respect to regulation of postnatal apoptosis in the cerebral cortex. Indeed, by engineering a mouse line in which a dominant-negative RhoA mutant (N19–RhoA) is specifically expressed in neurons, Sanno and colleagues have demonstrated that the inhibition of RhoA activity reduces the amount of apoptosis occurring in the postnatal cortex and results in a concomitant increase in the density and absolute number of neurons in the adult cortex (Sanno et al., 2010). Interestingly, the change in neuronal density in the N19–RhoA cortex is attributable to an increase in the number of excitatory projection neurons and not in that of the interneuron population, which originates in the ventral telencephalon (Sanno et al., 2010).

Besides the well-established programmed cell death naturally occurring in the postnatal brain, more recent studies indicate the existence of an earlier wave of programmed cell death affecting neural progenitors and nascent neurons (Yeo and Gautier, 2004). This early wave of cell death appears to play an even more critical role in determining the final size of the brain (de la Rosa and de Pablo, 2000; Kuan et al., 2000). In mouse, this death occurs between E12 and E16 within the VZ and IZ of the cerebral cortex (Blaschke et al., 1996, 1998; Thomaidou et al., 1997). Interestingly, the forebrain specific deletion of *Rac1* mediated by *FoxG1^Cre^* enhances apoptosis in VZ and SVZ progenitors, mainly around E14.5 (Chen et al., 2009; Leone et al., 2010); this effect partially contributes to a decrease in neural progenitors observed during mid-to-late telencephalic development (Chen et al., 2009). While Rac1 is required for survival of both VZ and SVZ progenitors, Cdc42 plays a dispensable role in cell survival during corticogenesis, as indicated by a comparable number of apoptotic cells in the cortex of control mice and *Cdc42/Nestin–Cre* knockout mutants (Peng et al., 2013).

## Upstream regulators of Rho GTPases and cortical projection neuron development

A number of *in vivo* studies showing involvement of GEFs and GAPs in cortical projection neuron development further support a critical role of the Rho GTPase family in this process and are summarized in Table 2. Studying the functions of GAPs and GEFs not only provides an indirect way to clarify the roles played by Rho GTPases in specific steps of corticogenesis, but it also contributes to the overall understanding of the entire pathways activated downstream of Rho proteins. For example, the loss of *NOMA-GAP*, a Cdc42-specific GAP, leads to an oversimplification of cortical dendritic arborization, as well as an hyperactivation of Cdc42. Remarkably, these dendritic defects can be partially restored by genetic reduction of post-mitotic *Cdc42* levels, demonstrating that the post-mitotic inhibition of Cdc42, mediated by NOMA-GAP, is a necessary requirement for dendritic branching during cortical development (Rosario et al., 2012). In this study, the authors further show that *in utero* expression of active cofilin is sufficient to restore postnatal dendritic complexity in *NOMA-GAP*-deficient animals. Therefore, these data support a model, whereby, during cortical dendritic development, cofilin activation is positively regulated by NOMA-GAP through the inhibition of Cdc42 (Rosario et al., 2012). Interestingly, only the dendritic complexity of layer II/III neurons is affected by the genetic ablation of *NOMA-GAP*, whereas layer V pyramidal neurons develop normal dendrites. How NOMA-GAP regulates dendrite development only in specific layers, although this GAP is expressed in all cortical layers (Rosario et al., 2012), is still an open question. It is possible that other Cdc42-GAPs, which are selectively expressed in the unaffected layers, might substitute for the loss of *NOMA-GAP* function. Alternatively, some neurons might be more sensitive to NOMA-GAP activity, because they exhibit higher active Cdc42 levels, which could be the result of layer-specific environmental signals or differentially expressed Cdc42-GEFs.

One difficulty with these studies is to determine whether the phenotype observed is due to the inhibition or activation of a specific Rho GTPase or of several members, since these regulators usually show activity against multiple Rho GTPases. For instance, RICS has been classified in Table 2 as a Rho GAP, because the defects on dendrites induced by *RICS* knockdown are rescued by an inhibition of RhoA signaling (Long et al., 2013) and might thus be due to a modulation of Rho activity. However, it has been shown that RICS prefers Cdc42 over Rac1 or RhoA as a substrate (Simo and Cooper, 2012). Also, it is important to remember that some of the phenotypes described in Table 2 might not be related to the GAP or GEF activity itself, but other protein domains might be involved. For example, α2-chimaerin, a Rac-GAP, has been shown to regulate radial migration, not through its GAP activity, which is instead dispensable, but through the association with the microtubule-associated protein CRMP-2 (Ip et al., 2012). Similarly, DOCK7 is a Rac-GEF that controls INM independently of its GEF activity (Yang et al., 2012b). The functions of GAPs and GEFs that are independent from GTPase activation or GDP-GTP exchange, respectively, have not been included in Table 2.

## Rho GTPases and cerebral cortex malformations

As described previously, the development of the cerebral cortex is remarkably complex and tightly organized. Disruption of any of the overlapping steps that contribute to this process can result in profound and stereotypical cortical malformations. In view of the multiple regulatory functions played by Rho GTPases during cerebral cortex development, it is thus not surprising that their forebrain specific suppression leads to cortical malformations. Accordingly, Cappello et al. found that *Emx1-Cre* mediated deletion of *RhoA* causes three types of malformations in the mouse cerebral cortex (Cappello et al., 2012; Cappello, 2013). First, the adult mutant cerebral cortex is about 1.3-fold thicker than control. This megalencephaly phenotype has been linked to the increased proliferation observed in the *RhoA* conditional mutant (see Section Rho GTPases and Regulation of Progenitor Cell Division, Proliferation and Cell Fate and Table 1). The second malformation observed in these mutants is a subcortical band heterotopia, which is characterized by a heterotopic cortex formed of ectopic neurons embedded within the white matter and underlying a normotopic cortex. Interestingly, the formation of this double cortex may not result from direct defects in migrating neurons, but rather from defective radial glia fibers that neurons use as a scaffold to migrate. Indeed, as mentioned previously (see Section Rho GTPases and Regulation of Radial Migration and Table 1), *RhoA* null neurons migrate normally when transplanted into wild-type cerebral cortex, whereas the converse is not the case, probably because of the strongly disorganized RG processes in the mutant cortices (Cappello et al., 2012). The last cortical abnormality observed in *Emx1-Cre RhoA* null mutants is the formation of cobblestones or neuronal ectopias at the basal side of the developing cerebral cortex. These neuronal protrusions beyond layer I may result from the increase speed of migrating nascent neurons and/or from the aberrant RG endfeets (Cappello, 2013). Interestingly, *FoxG1^Cre^ RhoA-*deficient embryos also exhibit expansion of the neural progenitor pool and exencephaly-like protrusions (Katayama et al., 2011).

In contrast to *RhoA* deletion, which induces excessive proliferation, *Rac1* loss of function in the mouse cerebral cortex reduces progenitor cell proliferation and increases apoptosis, which may be the major causes of microcephaly observed in the mutants (Chen et al., 2009; Leone et al., 2010). The suppression of *Cdc42* in the cortex also affects the overall cortical morphology. Indeed, *Cdc42*-deficient telencephalon fails to bulge or separate into two cerebral hemispheres, resulting in holoprosencephaly (Chen et al., 2006). This phenotype may result from the essential role of Cdc42 in establishing the apico-basal polarity of RGs and subsequently of the telencephalic neuroepithelium, which is needed for the expansion and bifurcation of cerebral hemispheres (Chen et al., 2006).

Interestingly, *NOMA-GAP* deficiency also leads to a decrease of cortical thickness in different cortical areas (Rosario et al., 2012). This reduction was observed in adult mice, but also at early postnatal stages, suggesting that cortical thinning is due to a defective developmental process. Since the absence of *NOMA-GAP* does not impact the early stages of cortical development, including neuronal birth, survival, fate determination and migration, cortical thinning in these mutants may arise from defective formation of cortical dendritic trees (Rosario et al., 2012).

## Concluding remarks

Altogether the above studies highlight the crucial roles played by the Rho GTPase family in the regulation of cerebral cortex development and emphasize that a better understanding of these functions might help to clarify the etiology of several cortical malformations. Further work is required to fully characterize the contribution of the different Rho GTPases expressed in the developing cerebral cortex as well as the downstream signaling involved and the mechanisms regulating their expression and/or activity. Another major challenge for the future will be to understand how the signals through the different Rho GTPases as well as the other small GTPases of the Ras superfamily (Ras, Rab, Ran, Arf) are integrated in nascent cortical neurons and how they are spatially and temporally controlled during cortical development.

Abnormal signaling through Rho GTPases is associated with cognitive dysfunction (Newey et al., 2005; De Filippis et al., 2014) and recent findings have showed their involvement in the development and progression of neurodegenerative diseases (Antoine-Bertrand et al., 2011). Studies on Rho GTPase functions *in vivo* thus represent an underexplored territory that may hold therapeutic potential.

### Conflict of interest statement

The authors declare that the research was conducted in the absence of any commercial or financial relationships that could be construed as a potential conflict of interest.
